# Association of *LTF*, *ENAM*, and *AMELX* polymorphisms with dental caries susceptibility: a meta-analysis

**DOI:** 10.1186/s12903-020-01121-7

**Published:** 2020-05-06

**Authors:** Roohollah Sharifi, Sajjad Jahedi, Hamid Reza Mozaffari, Mohammad Moslem Imani, Masoud Sadeghi, Amin Golshah, Hedaiat Moradpoor, Mohsen Safaei

**Affiliations:** 1 Department of Endodontics, School of Dentistry, Kermanshah University of Medical Sciences, Kermanshah, 6713954658 Iran; 2 Students Research Committee, Kermanshah University of Medical Sciences, Kermanshah, 6715847141 Iran; 3 Department of Oral and Maxillofacial Medicine, School of Dentistry, Kermanshah University of Medical Sciences, Kermanshah, 6713954658 Iran; 4 Department of Orthodontics, School of Dentistry, Kermanshah University of Medical Sciences, Kermanshah, 6713954658 Iran; 5 Medical Biology Research Center, Kermanshah University of Medical Sciences, Kermanshah, 6714415185 Iran; 6 Department of Prosthodontics, School of Dentistry, Kermanshah University of Medical Sciences, Kermanshah, 6713954658 Iran; 7 Advanced Dental Sciences Research Laboratory, School of Dentistry, Kermanshah University of Medical Sciences, Kermanshah, 6713954658 Iran

**Keywords:** Dental caries, Polymorphism, Lactotransferrin, Enamelin, Amelogenin X

## Abstract

**Background:**

This meta-analysis evaluated the association of *LTF*, *ENAM*, and *AMELX* polymorphisms with dental caries susceptibility.

**Methods:**

We searched the Scopus, PubMed/Medline, Web of Science, and Cochrane Library databases to retrieve articles published by October 2019. Review Manager 5.3 software was used to estimate the odds ratios (ORs) and 95% confidence intervals (CIs). The results of publication bias tests were retrieved by Comprehensive Meta-Analysis 2.0 software.

**Results:**

A total of 150 relevant records were identified; out of which, 16 were entered into the analysis (4 studies assessed *LTF*, 11 *ENAM*, and 11 *AMELX* polymorphisms). Of all polymorphisms, there was a significant association only between *ENAM* rs3796704 polymorphism and dental caries susceptibility. Both *ENAM* rs3796704 and *AMELX* rs17878486 polymorphisms had a significant association with dental caries risk in the Caucasian ethnicity and the studies including caries-free control group.

**Conclusions:**

The results of this meta-analysis showed that the G allele and the GG genotype of *ENAM* rs3796704 were associated with an increased risk of caries in the case group compared with the control group. But there was no association between *LTF* rs1126478, *ENAM* (rs1264848 and rs3796703), and *AMELX* (rs946252, rs17878486, and rs2106416) polymorphisms and dental caries susceptibility.

## Highlights

1. There was an association between *ENAM* rs3796704 polymorphism and the risk of dental caries.

2. There was no association between polymorphisms of *LTF* (rs1126478), *ENAM* (rs1264848 and rs3796703), and *AMELX* (rs946252, rs17878486, and rs2106416) and dental caries susceptibility.

## Introduction

Dental caries can significantly affect the general health and quality of life in the modern world [1]. Dental caries develops following demineralization of tooth structure and often results in pulpal and periapical inflammation, and subsequent pain, infection and tooth loss [1, 2]. Some environmental conditions and population groups may contribute to higher incidence of dental caries [2]. Environmental risk factors may also affect dental caries development [3]. Although exposed to the same environmental conditions, some patients may be more sensitive or more resistant to dental caries than others; such differences may be due to genetic factors in dental caries etiopathogenesis [4]. New findings raised possibilities of presence of associations between genetic factors and dental caries [5]. The etiology of dental caries involves complex interactions between genetic and environmental factors. Three prevalent genes are reportedly involved in development of dental caries namely the *lactotransferrin (LTF)*, *enamelin (ENAM)*, and *amelogenin X (AMELX)*. The rs1126478 polymorphism of *LTF* (a saliva protein gene) produces a shift from arginine to lysine at amino acid position 47 in the antimicrobial region, and presents transcriptional activation activity [6]. *LTF* can decrease the lipopolysaccharide-activated innate immune response, regulate the adaptive immune system [7], and play a significant role in physiological homoeostasis, which is in turn related to disease development [8]. *ENAM* (a member of P/Q-rich secretory calcium-binding phosphoprotein cluster genes) is located on chromosome 4q 13.3 [9]. The gene encodes the protein enamelin, which is the largest protein found in the enamel matrix and is involved in dental enamel mineralization and its structural organization [10]. *AMELX* is an essential gene that produces amelogenin as the main protein of dental enamel during the secretion stage of amelogenesis [11]. *AMELX* polymorphisms result in distinct alternations in enamel microstructure [12]. Therefore, these polymorphisms play a critical role in regulation of mineralization and enamel thickness [13]. The association between the mutations of *LTF*, *ENAM*, and *AMELX* genes and dental caries susceptibility has been shown in some studies [5, 14, 15]. Therefore, we aimed to assess the association of polymorphisms of these genes and the risk of dental caries in a meta-analysis of case-control studies and therefore evaluating only the genetic influences for dental caries.

## Materials and methods

### Search strategy and study selection

One author systematically searched the PubMed/Medline, Web of Science, Cochrane Library, and Scopus databases to retrieve articles published by October 2019 without publication period, language, and patient’s age restrictions. The search terms or keywords were (“lactotransferrin” or “lactoferrin” or “LTF” or “enamelin” or “ENAM” or “amelogenin X” or “AMELX”) and (“dental caries” or “caries” or “decay”) and (“gene” or “polymorphism” or “variant” or “genetic”). In addition, the references of the retrieved articles related to the topic including original and review articles were searched to make sure that no study was missed. After article retrieval, another author assessed the titles and abstracts of the articles related to the topic; subsequently, the full-texts of the articles that met our eligibility criteria were downloaded and screened. After screening, the exclusion reason was recorded for any study removed, and the disagreements between the authors were resolved by another author.

### Eligibility criteria

The inclusion criteria were as follows: (I) studies including two independent groups (case group with caries or high caries and caries-free control group or with low/very low caries) without age restriction, (II) studies with any defined Decayed, Missing, and Filled Teeth (DMFT) score for the two groups, (III) studies including one or more polymorphisms of *LTF*, *ENAM*, and *AMELX* genes with a minimum of two relevant studies for the analysis; for example, four studies assessed *LTF* rs1126478, *ENAM* rs1264848, *ENAM* rs3796704, *ENAM* rs3796703, *AMELX* rs946252, *AMELX* rs17878486, *AMELX* rs6639060, and *AMELX* rs2106416 polymorphisms; and (IV) patients and controls had to have no genetic diseases, chronic illnesses, or other disorders. We excluded irrelevant studies, studies without sufficient data for analysis, studies without a control group, studies including less than 20 individuals in each group, duplicate studies, animal studies, case reports, conference papers, reviews, and systematic reviews.

### Data abstraction

Two authors independently abstracted the data of the studies analyzed in the meta-analysis. The data from each study, including first author, publication year, country of residence of the included individuals, ethnicity, mean age of individuals in the two groups, age group of individuals in each study, genotyping method, DMFT score of the two groups, and type of reported polymorphism (s) in each study, were extracted and analyzed.

### Statistical analysis

Review Manager 5.3 (RevMan 5.3) software was applied to compute the odds ratios (ORs) and 95% confidence intervals (CIs). To estimate the significance of the pooled OR by the Z test, a *p*-value (two-sided) < 0.05 was considered significant. The I^2^ statistic was used to estimate heterogeneity. A *p* < 0.1 or I^2^ > 50% indicated a significant heterogeneity and we used the random-effects model for such cases; if not, the fixed-effects model was used. The publication bias across the studies was assessed using the Egger’s and Begg’s tests. If *p* < 0.05 (two-sided) for both tests or one, there was a significant degree of publication bias. In order to evaluate the stability/consistency of the results, the sensitivity analysis with both “the removal of one study” and “cumulative analysis” was performed. The results of these tests were retrieved by Comprehensive Meta-Analysis 2.0 (CMA 2.0) software. All authors revised the extracted data and the analyses and the disagreement between them was resolved by a discussion.

## Results

### Study selection

**Fig. 1:**
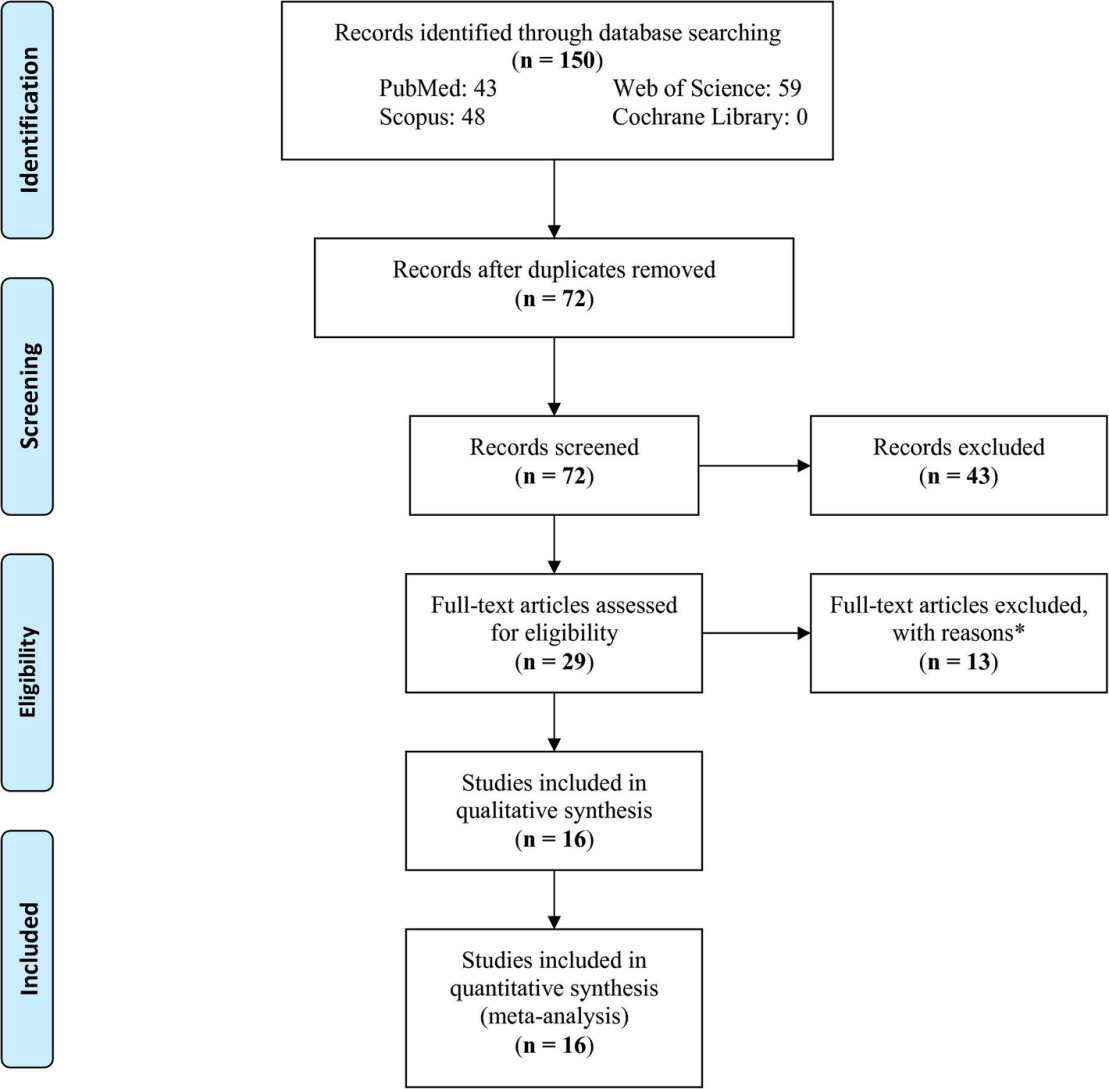
Flow chart of study selection

A total of 150 records were identified in the databases; after removing the duplicates and irrelevant records, 29 full-text articles were evaluated for eligibility (Fig. 1). Next, 13 articles were excluded with reasons: two studies were systematic reviews, two studies were reviews, three studies lacked sufficient data, two studies had no control group, three studies did not report any of the mentioned polymorphisms in this meta-analysis and did not have any known polymorphism either, and one study reported rs1126478 polymorphism with less than 20 individuals in each group. Finally, 16 studies were entered into the analysis.

### Study characteristics

The characteristics of 16 studies included in this meta-analysis are presented in Table 1. The articles had been published from 2008 to 2019. Eleven studies had been conducted on Caucasians [12, 14–16, 18–23, 25], four studies had been conducted on Asians [5, 13, 17, 24], and one on mixed [11] ethnicities. Eleven studies evaluated children [5, 11, 13–16, 18–20, 22, 24], three studies evaluated adults [17, 21, 23], and two studies evaluated both adults and children [12, 25]. Four studies had assessed *LTF* rs1126478 in 1556 cases and 1106 controls [5, 16, 20, 24]. Five studies had assessed *ENAM* rs1264848 in 934 cases and 600 controls [11, 14, 19, 22, 25]. Four studies had assessed *ENAM* rs3796704 in 574 cases and 533 controls [11, 19, 23, 25]. Two studies had assessed *ENAM* rs3796703 in 585 cases and 567 controls [5, 13]. Three studies had assessed *AMELX* rs946252 in 151 cases and 147 controls [11, 12, 19]. Four studies had assessed *AMELX* rs17878486 in 249 cases and 193 controls [11, 15, 17, 19]. Two studies had assessed *AMELX* rs6639060 in 157 cases and 144 controls [13, 21], and two studies had assessed *AMELX* rs2106416 in 175 cases and 110 controls [13, 18]. One study [18] included two subsets. The genotyping method in all studies was based on polymerase chain reaction (PCR). The controls in six studies [11, 17, 18, 21–23] had low/very low rate of dental caries and others were caries-free. In addition, the two groups in the studies were introduced as the caries group versus the caries-free group, or the high-caries group versus the low/very low caries group.

**Table 1:** Characteristics of the studies included in this meta-analysis

First author, publication year	Country	Ethnicity	Mean age, years (Case/ Control)	Age group of individuals	Genotyping method	DMFT score (Case/Control)	Polymorphisms
Ouryouji, 2008 [13]	Japan	Asian	5.4/4.8	Children	PCR-RFLP	≥10/0	*ENAM* rs3796703 *AMELX* rs6639060 *AMELX* rs2106416
Azevedo, 2010 [16]	Brazil	Caucasian	12	Children	PCR-SSCP	≥1/0	*LTF* rs1126478
Kang, 2011^a^ [17]	Korea	Asian	21.3/23.2	Adults	PCR	> 2/≤2	*AMELX* rs17878486
Olszowski, 2012^ab^ [18]	Poland	Caucasian	5	Children	PCR-RFLP	≥3/< 3	*AMELX* rs2106416
			13	Children	PCR-RFLP	≥3/< 3	*AMELX* rs2106416
Gasse, 2013 [12]	France	Caucasian	7.6/22	Both	PCR	≥4/0	*AMELX* rs946252
Jeremias, 2013^a^ [11]	Brazil	Mixed	Case: < 6 Control: < 20	Children	RT-PCR	≥4/≤3	*ENAM* rs1264848 *ENAM* rs3796704 *AMELX* rs946252 *AMELX* rs17878486
Ergöz, 2014 [19]	Turkey	Caucasian	8.7/8.7	Children	TaqMan	≥1/0	*ENAM* rs1264848 *ENAM* rs3796704 *AMELX* rs946252 *AMELX* rs17878486
Volckova, 2014 [20]	Czech	Caucasian	Range: 11–13	Children	PCR-RFLP	≥1/0	*LTF* rs1126478
Gerreth, 2016 [14]	Poland	Caucasian	2.6	Children	RT-PCR	≥1/0	*ENAM* rs1264848
Yildiz, 2016^a^ [21]	Turkey	Caucasian	Range: 20–60	Adults	PCR-RFLP	≥14/≤5	*AMELX* rs6639060
Gerreth, 2017 [15]	Poland	Caucasian	Range: 1.7–3.5	Children	TaqMan	≥1/0	*AMELX* rs17878486
Wang, 2017 [5]	China	Asian	3.5/3.7	Children	PCR	≥4/0	*LTF* rs1126478 *ENAM* rs3796703
Borilova Linhartova, 2018^a^ [22]	Czech	Caucasian	Range: 13–15	Children	TaqMan	≥1/0	*ENAM* rs1264848
Koohpeima, 2018^a^ [23]	Iran	Caucasian	29.8/28.4	Adults	ARMS-PCR	NA	*ENAM* rs3796704
Wang, 2018 [24]	China	Asian	Range: 2–4	Children	PCR	≥8/0	*LTF* rs1126478
Devang Divakar, 2019 [25]	Saudi Arabia	Caucasian	6.9/23.2	Both	RT-PCR	≥4/0	*ENAM* rs1264848 *ENAM* rs3796704

^a^Case group included individuals with high caries and control group included individuals with low/very low caries. ^b^ This study had two subsets: one subset was reported in 5-year-olds and another in 13-year-olds

Abbreviations: *NA* Not available, *PCR* Polymerase chain reaction, *RFLP* Restriction fragment length polymorphism, *RT* Real time, *ARMS* Amplification refractory mutation system, *LTF* Lactotransferrin, *ENAM* Enamelin; AMELX; Amelogenin X

The genotype prevalence of each polymorphism included in each study in both case and control groups and the *p*-value for the Hardy-Weinberg equilibrium (HWE) for the controls are shown in Table 2. The controls in three studies [11, 12, 21] had a deviation from the HWE (*P* < 0.05).

**Table 2:** Prevalence of genotypes of the three polymorphisms (*LTF*, *ENAM* and *AMELX*)

First author, publication year	*LTF* rs1126478^a^		*ENAM* s1264848^a^		*ENAM* rs3796704^a^		*ENAM* rs3796703^b^		P-value of HWE for controls
	Case	Control	Case	Control	Case	Control	Case	Control	
Ouryouji, 2008 [13]	NA	NA	NA	NA	NA	NA	76/4/0	59/8/0	0.603
Azevedo, 2010 [16]	16/30/16	19/22/7	NA	NA	NA	NA	NA	NA	0.877
Jeremias, 2013 [11]	NA	NA	13/44/20	21/41/20	4/11/61	1/19/57	NA	NA	0.999/0.676
Ergöz, 2014 [19]	NA	NA	4/41/55	1/35/64	0/16/78	1/27/69	NA	NA	0.108/0.351
Volckova, 2014 [20]	288/150/44	86/56/13	NA	NA	NA	NA	NA	NA	0.374
Gerreth, 2016 [14]	NA	NA	8/37/3	4/26/18	NA	NA	NA	NA	0.202
Wang, 2017 [5]	64/222/219	64/209/227	NA	NA	NA	NA	439/64/2	458/42/0	0.149/0.327
Borilova Linhartova, 2018 [22]	NA	NA	45/259/237	19/74/84	NA	NA	NA	NA	0.656
Koohpeima, 2018 [23]	NA	NA	NA	NA	0/32/204	0/20/146	NA	NA	0.409
Wang, 2018 [24]	63/224/220	51/168/184	NA	NA	NA	NA	NA	NA	0.196
Devang Divakar, 2019 [25]	NA	NA	89/58/21	116/68/9	11/58/99	25/76/92	NA	NA	0.809/0.146
First author, publication year	*AMELX* rs946252^b^		*AMELX* rs17878486^b^		*AMELX* rs6639060^b^		*AMELX* rs2106416^b^		*P*-value of HWE for controls
	Case	Control	Case	Control	Case	Control	Case	Control	
Ouryouji, 2008 [13]	NA	NA	NA	NA	80/0/0	67/0/0	78/2/0	16/6/0	NA/0.458
Kang, 2011 [17]	NA	NA	1/2/82	0/2/29	NA	NA	NA	NA	0.852
Olszowski, 2012 (i) [18]	NA	NA	NA	NA	NA	NA	24/13/0	21/12/1	0.643
Olszowski, 2012 (ii) [18]	NA	NA	NA	NA	NA	NA	26/26/6	28/20/2	0.495
Gasse, 2013 [12]	5/9/25	4/1/25	NA	NA	NA	NA	NA	NA	**< 0.0001**
Jeremias, 2013 [11]	61/12/5	63/13/5	4/15/57	15/11/56	NA	NA	NA	NA	**0.002/< 0.0001**
Ergöz, 2014 [19]	5/18/11	10/17/9	8/18/14	3/21/8	NA	NA	NA	NA	0.742/0.051
Yildiz, 2016 [21]	NA	NA	NA	NA	54/11/12	56/9/12	NA	NA	**< 0.0001**
Gerreth, 2017 [15]	NA	NA	8/10/30	31/14/3	NA	NA	NA	NA	0.422

Abbreviations: *LTF* Lactotransferrin, *ENAM* Enamelin, *AMELX* Amelogenin X, *NA* Not available, *HWE* Hardy-Weinberg equilibrium

^a^Genotypes: AA/AG/GG

^b^Genotypes: CC/CT/TT. i: 5-year-olds. ii: 13-year-olds

### Pooled analysis

Table 3 shows the results of pooled analysis of each polymorphism based on five genetic models. Among the polymorphisms, only the G allele [OR = 1.38; 95%CI: 1.08, 1.76; *P =* 0.009; I^2^ = 27% (P_heterogeneity_ or P_h_ = 0.25)] and the GG genotype [OR = 1.41; 95%CI: 1.06, 1.87; *P* = 0.02; I^2^ = 18, (P_h_ = 0.30)]) of *ENAM* rs3796704 had an elevated risk in the case group compared with the control group. In addition, the funnel plots of each polymorphism are presented in the Supplementary file. Therefore, there was a significant association between *ENAM* rs3796704 polymorphism and dental caries susceptibility.

**Table 3:** Results of pooled analysis of each polymorphism based on five genetic models

Polymorphism, (number of studies)	G vs. A	GG vs. AA	AG vs. AA	AG + GG vs. AA	GG vs. AA + AG
	OR (95%CI), *P*-value, I^2^ (%), P_h_	OR (95%CI), *P*-value, I^2^ (%), P_h_	OR (95%CI), *P*-value, I^2^ (%), P_h_	OR (95%CI), *P*-value, I^2^ (%), P_h_	OR (95%CI), *P*-value, I^2^ (%), P_h_
*LTF* rs1126478 (*n* = 4)	0.98 (0.87, 1.10), 0.68, 25, 0.26	1.03 (0.80, 1.33), 0.82, 4, 0.37	1.00 (0.80, 1.25), 0.99, 4, 0.44	0.99 (0.81, 1.23), 0.96, 7, 0.36	0.95 (0.80, 1.13), 0.58, 0, 0.45
*ENAM* rs1264848 (*n* = 5)	0.93 (0.65, 1.32), 0.68, 75, 0.003	0.88 (0.34, 2.30), 0.79, 76, 0.002	1.21 (0.88, 1.65), 0.24, 0, 0.48	1.24 (0.93, 1.67), 0.15, 24, 0.26	0.83 (0.44, 1.58), 0.58, 79, 0.0007
*ENAM* rs3796704 (n = 4)	**1.38 (1.08, 1.76), 0.009, 27, 0.25**	1.86 (0.96, 3.62), 0.07, 44, 0.17	1.27 (0.64, 2.52), 0.49, 50, 0.13	1.64 (0.86, 3.14), 0.13, 43, 0.17	**1.41 (1.06, 1.87), 0.02, 18, 0.30**
Polymorphism, (number of studies)	T vs. C	TT vs. CC	CT vs. CC	CT + TT vs. CC	TT vs. CC + CT
	OR (95%CI), *P*-value, I^2^ (%), P_h_	OR (95%CI), *P*-value, I^2^ (%), P_h_	OR (95%CI), *P*-value, I^2^ (%), P_h_	OR (95%CI), *P*-value, I^2^ (%), P_h_	OR (95%CI), *P*-value, I^2^ (%), P_h_
*ENAM* rs3796703 (*n* = 2)	0.92 (0.24, 3.58), 0.91, 78, 0.03	5.22 (0.25, 108.96), 0.29	0.89 (0.23, 3.48), 0.87, 77, 0.04	0.90 (0.23, 3.63), 0.89, 78, 0.03	2.90 (0.92, 9.12), 0.07, 0, 0.69
*AMELX* rs946252 (*n* = 3)	1.01 (0.68, 1.51), 0.95, 39, 0.20	1.27 (0.58, 2.75), 0.55, 0, 0.51	1.45 (0.75, 2.81), 0.27, 31, 0.23	1.21 (0.68, 2.14), 0.52, 0, 0.51	0.82 (0.43, 1.56), 0.54, 0.38, 0.20
*AMELX* rs17878486 (n = 4)	2.25 (0.81, 6.24), 0.12, 86, 0.0001	3.59 (0.55, 23.32), 0.18, 81, 0.001	1.45 (0.36, 5.80), 0.60, 0.67, 0.03	2.30 (0.51, 10.33), 0.28, 77, 0.004	3.10 (0.85, 11.28), 0.09, 81, 0.001
*AMELX* rs6639060 (n = 2)	1.08 (0.63, 1.85), 0.78	1.04 (0.43, 2.51), 0.94	1.27 (0.49, 3.30), 0.63	1.14 (0.56, 2.29), 0.72	1.00 (0.42, 2.39), 1.00
*AMELX* rs2106416 (n = 3)	0.59 (0.16, 2.11), 0.41, 82, 0.003	1.83 (0.47, 7.06), 0.38, 40, 0.20	0.55 (0.13, 2.25), 0.40, 80, 0.006	0.59 (0.15, 2.31), 0.45, 79, 0.008	1.67 (0.44, 6.34), 0.45, 31, 0.23

Abbreviations: *LTF* Lactotransferrin, *ENAM* Enamelin, *AMELX* Amelogenin X, *NA* Not available, *OR* Odds ratio, *CI* Confidence interval; P_h_, P_heterogeneity_

### Subgroup analysis

Table 4 identifies the subgroup analyses based on the ethnicity, age group, and the control group for each polymorphism and in five genetic models. The results showed that the G allele and the GG and AG genotypes of *ENAM* rs3796704 had an elevated risk in the case group compared with the control group in the Caucasian ethnicity; whereas in studies with a caries-free control group, the G allele and GG genotype had an elevated risk in the case group compared with the control group. In addition, the T allele, and TT and CT genotypes of *AMELX* rs17878486 polymorphism had an elevated risk in the case group compared with the control group in mixed ethnicity and studies with a caries-free control group. Therefore, there was a significant association between both polymorphisms of *ENAM* rs3796704 and *AMELX* rs17878486 and dental caries susceptibility in the Caucasian ethnicity and studies including caries-free individuals as the control group.

**Table 4:** Subgroup analysis of each polymorphism based on ethnicity, age group, and control group

Polymorphism, (number of studies)	G vs. A	GG vs. AA	AG vs. AA	AG + GG vs. AA	GG vs. AA + AG
	OR (95%CI), *P*-value, I^2^ (%), P_h_	OR (95%CI), *P*-value, I^2^ (%), P_h_	OR (95%CI), *P*-value, I^2^ (%), P_h_	OR (95%CI), *P*-value, I^2^ (%), P_h_	OR (95%CI), *P*-value, I^2^ (%), P_h_
LTF rs1126478					
Ethnicity					
Caucasian (2)	1.18 (0.66, 2.11), 0.58, 73, 0.05	1.49 (0.58, 3.85), 0.41, 55, 0.13	1.02 (0.53, 1.97), 0.95, 53, 0.14	1.16 (0.53, 1.97), 0.95, 0.53, 0.14	1.33 (0.77, 2.29), 0.30, 6, 0.30
Asian (2)	0.96 (0.84, 1.09), 0.52, 0, 0.98	0.97 (0.73, 1.29), 0.81, 0, 0.99	1.07 (0.80, 1.43), 0.64, 0, 0.96	1.02 (0.78, 1.33), 0.91, 0, 0.97	0.92 (0.77, 1.10), 0.35, 0, 0.96
Age group					
Children (4)	0.98 (0.87, 1.10), 0.68, 25, 0.26	1.03 (0.80, 1.33), 0.82, 4, 0.37	1.00 (0.80, 1.25), 0.99, 4, 0.44	0.99 (0.81, 1.23), 0.96, 7, 0.36	0.95 (0.80, 1.13), 0.58, 0, 0.45
Control group					
Caries free (4)	0.98 (0.87, 1.10), 0.68, 25, 0.26	1.03 (0.80, 1.33), 0.82, 4, 0.37	1.00 (0.80, 1.25), 0.99, 4, 0.44	0.99 (0.81, 1.23), 0.96, 7, 0.36	0.95 (0.80, 1.13), 0.58, 0, 0.45
ENAM rs1264848					
Ethnicity					
Caucasian (4)	0.86 (0.56, 1.32), 0.49, 80, 0.002	0.67 (0.18, 2.45), 0.54, 82, 0.0009	1.13 (0.80, 1.59), 0.48, 0, 0.45	1.18 (0.86, 1.62), 0.31, 35, 0.20	0.76 (0.34, 1.72), 0.51, 84, 0.0003
Mixed (1)	1.23 (0.79,1.91), 0.36	1.62 (0.64, 4.09), 0.31	1.73 (0.77, 3.90), 0.18	1.69 (0.78, 3.68), 0.18	1.09 (0.53, 2.23), 0.82
Age group					
Children (4)	0.82 (0.57, 1.18), 0.28, 67, 0.03	0.58 (0.19, 1.82), 0.35, 74, 0.01	1.30 (0.84, 2.01), 0.26, 3, 0.36	1.16 (0.76, 1.75), 0.49, 42, 0.16	0.66 (0.37, 1.17), 0.15, 70, 0.02
Control group					
Caries free (3)	0.79 (0.39, 1.63), 0.53, 86, 0.0006	0.42 (0.03, 5.41), 0.51, 88, 0.0003	1.00 (0.66, 1.51), 1.00, 0, 0.44	0.74 (0.27, 2.02), 0.56, 55, 0.11	0.66 (0.15, 2.95), 0.59, 89, < 0.0001
Low caries (2)	1.03 (0.83, 1.29), 0.78, 0, 0.37	1.30 (0.79, 2.14), 0.30, 0, 0.59	1.57 (0.97, 2.53), 0.07, 0, 0.76	1.45 (0.92, 2.28), 0.11, 0, 0.62	0.90 (0.66, 1.23), 0.51, 0, 0.57
ENAM rs3796704					
Ethnicity					
Caucasian (3)	**1.43 (1.10, 1.85), 0.007, 45, 0.16**	**2.49 (1.19, 5.24), 0.02, 0, 0.85**	1.74 (0.81, 3.74), 0.16, 0, 0.98	**2.16 (1.05, 4.45), 0.04, 0, 0.85**	**1.41 (1.04, 1.91), 0.03, 45, 0.16**
Mixed (1)	1.11 (0.57, 2.15), 0.77	0.27 (0.03, 2.47), 0.24	0.14 (0.01, 1.46), 0.10	0.24 (0.03, 2.17), 0.20	1.43 (0.67, 3.05), 0.36
Age group					
Children (2)	1.46 (0.92, 2.32), 0.11, 22, 0.26	0.61 (0.13, 2.93), 0.54, 38, 0.20	0.35 (0.07, 1.74), 0.20, 35, 0.22	0.54 (0.11, 2.56), 0.44, 37, 0.21	**1.71 (1.02, 2.85), 0.04, 0, 0.53**
Control group					
Caries free (2)	**1.62 (1.21, 2.17), 0.001, 0, 0.59**	**2.499 (1.19, 5.24), 0.02, 0, 0.85**	1.74 (0.81, 3.74), 0.16, 0, 0.98	**2.16 (1.05, 4.45), 0.04, 0, 0.85**	**1.67 (1.17, 2.39), 0.0050, 0.58**
Low caries (2)	0.97 (0.63, 1.50), 0.89, 0, 0.61	0.27 (0.03, 2.47), 0.24	0.14 (0.01, 1.46), 0.10	0.24 (0.03, 2.17), 0.20	1.05 (0.66, 1.68), 0.83, 0, 0.32
Polymorphism, (number of studies)	T vs. C	TT vs. CC	CT vs. CC	CT + TT vs. CC	TT vs. CC + CT
	OR (95%CI), *P*-value, I^2^ (%), P_h_	OR (95%CI), *P*-value, I^2^ (%), P_h_	OR (95%CI), *P*-value, I^2^ (%), P_h_	OR (95%CI), *P*-value, I^2^ (%), P_h_	OR (95%CI), *P*-value, I^2^ (%), P_h_
ENAM rs3796703					
Ethnicity					
Asian (2)	0.92 (0.24, 3.58), 0.91, 78, 0.03	5.22 (0.25, 108.96), 0.29	0.89 (0.23, 3.48), 0.87, 77, 0.04	0.90 (0.23, 3.63), 0.89, 78, 0.03	2.90 (0.92, 9.12), 0.07, 0, 0.69
Age group					
Children (2)	0.92 (0.24, 3.58), 0.91, 78, 0.03	5.22 (0.25, 108.96), 0.29	0.89 (0.23, 3.48), 0.87, 77, 0.04	0.90 (0.23, 3.63), 0.89, 78, 0.03	2.90 (0.92, 9.12), 0.07, 0, 0.69
AMELX rs946252					
Ethnicity					
Caucasian (2)	0.95 (0.35, 2.55), 0.92, 69, 0.07	1.42 (0.54, 3.76), 0.48, 17, 0.27	2.80 (0.93, 8.43), 0.07, 0, 0.38	1.63 (0.67, 4.01), 0.28, 0, 0.42	0.73 (0.19, 2.87), 0.66, 67, 0.08
Mixed (1)	0.99 (0.53, 1.86), 0.98	1.03 (0.28, 3.75), 0.96	0.95 (0.40, 2.25), 0.91	0.98 (0.46, 2.07), 0.95	1.04 (0.29, 3.75), 0.95
Age group					
Children (2)	1.21 (0.77,1.91), 0.42, 0, 0.37	1.54 (0.61, 3.92), 0.36, 0, 0.37	1.24 (0.61, 2.49), 0.56, 5, 0.31	1.24 (0.66, 2.33), 0.50, 24, 0.25	1.26 (0.56, 2.83), 0.25
Control group					
Caries free (2)	0.95 (0.35, 2.55), 0.92, 69, 0.07	1.42 (0.54, 3.76), 0.48, 17, 0.27	2.80 (0.93, 8.43), 0.07, 0, 0.38	1.63 (0.67, 4.01), 0.28, 0, 0.42	0.73 (0.19, 2.87), 0.66, 67, 0.08
Low caries (1)	0.99 (0.53, 1.86), 0.98	1.03 (0.28, 3.75), 0.96	0.95 (0.40, 2.25), 0.91	0.98 (0.46, 2.07), 0.95	1.04 (0.29, 3.75), 0.95
AMELX rs17878486					
Ethnicity					
Caucasian (2)	2.87 (0.35, 23.23), 0.32, 95, < 0.0001	5.12 (0.09, 278.85), 0.42, 93, 0.0002	1.00 (0.12, 8.23), 1.00, 0.81, 0.02	2.03 (0.10, 42.44), 0.65, 92, 0.0004	6.17 (0.41, 92.05), 0.19, 91, 0.001
Asian (1)	1.38 (0.25, 7.75), 0.71	0.93 (0.04, 23.52), 0.97	0.33 (0.01, 12.82), 0.56	0.89 (0.04, 22.53), 0.95	1.89 (0.30, 11.85), 0.50
Mixed (1)	**1.87 (1.06, 3.30), 0.03**	**3.82 (1.19, 12.21), 0.02**	**5.11 (1.33, 19.72), 0.02**	**4.03 (1.27, 12.75), 0.02**	1.39 (0.69, 2.80), 0.35
Age group					
Children (3)	2.48 (0.76, 8.05), 0.13, 90, < 0.0001	4.69 (0.55, 40.16), 0.16, 86, 0.0007	1.72 (0.37, 7.92), 0.48, 75, 0.02	2.65 (0.49, 14.35), 0.26, 84, 0.002	3.55 (0.72, 17.60), 0.12, 87, 0.0004
Adults (1)	1.38 (0.25, 7.75), 0.71	0.93 (0.04, 23.52), 0.97	0.33 (0.01, 12.82), 0.56	0.89 (0.04, 22.53), 0.95	1.89 (0.30, 11.58), 0.50
Control group					
Caries free (2)	2.87 (0.35, 23.23), 0.32, 95, < 0.0001	5.12 (0.09, 278.85), 0.42, 93, 0.0002	1.00 (0.12, 8.23), 1.00, 0.81, 0.02	2.03 (0.10, 42.44), 0.65, 92, 0.0004	6.17 (0.41, 92.05), 0.19, 91, 0.001
Low caries (2)	**1.82 (1.06, 3.11), 0.03, 0, 0.74**	**3.28 (1.14, 9.43), 0.03, 0, 0.42**	**3.42 (1.04, 11.28), 0.04, 47, 0.17**	**3.43 (1.21, 9.75), 0.02, 0, 0.39**	1.44 (0.75, 2.77), 0.27, 0, 0.76
AMELX rs6639060					
Ethnicity					
Caucasian (1)	1.08 (0.63, 1.85), 0.78	1.04 (0.43, 2.51), 0.94	1.27 (0.49,3.30), 0.63	1.14 (0.56, 2.29), 0.72	1.00 (0.42, 2.39), 1.00
Age group					
Adults (1)	1.08 (0.63, 1.85), 0.78	1.04 (0.43, 2.51), 0.94	1.27 (0.49,3.30), 0.63	1.14 (0.56, 2.29), 0.72	1.00 (0.42, 2.39), 1.00
AMELX rs2106416					
Ethnicity					
Caucasian (2)	1.25 (0.77, 2.03), 0.37, 30, 0.23	1.83 (0.47, 7.06), 0.38, 40, 0.20	1.20 (0.65, 2.22), 0.56, 0, 0.54	1.25 (0.69, 2.27), 0.46, 0, 0.35	1.67 (0.44, 6.34), 0.45, 31, 0.23
Age group					
Children (3)	0.59 (0.16, 2.11), 0.41, 82, 0.003	1.83 (0.47, 7.06), 0.38, 40, 0.20	0.55 (0.13, 2.25), 0.40, 80, 0.006	0.59 (0.15, 2.31), 0.45, 79, 0.008	1.67 (0.44, 6.34), 0.45, 31, 0.23

Abbreviations: *LTF* Lactotransferrin, *ENAM* Enamelin, *AMELX* Amelogenin X, *OR* Odds ratio, *CI* Confidence interval; P_h_, P_heterogeneity_

### Sensitivity analysis

One study [19] was omitted from the analysis of *AMELX* rs17878486 because the outlier data and the results illustrated that by deleting this study, the CT [OR = 3.07; 95%CI: 1.36, 6.94; *P* = 0.007; I^2^ = 0% (P_h_ = 0.37)] and CT + TT [OR = 5.72; 95%CI: 2.83, 11.59; P = < 0.00001; I^2^ = 21% (P_h_ = 0.0.28)] genotypes in dental caries patients were significantly superior to controls and with a low heterogeneity, respectively (Table 3)**.** In addition, other sensitivity analyses including “one study excluded” and “cumulative analysis” were performed and the previous results did not change qualitatively. Although the genotype distribution of the controls in three studies [11, 12, 21] did not follow the HWE, these analyses reported that the pooled ORs based on all genetic models were steady.

### Publication bias

**Fig. 2:**
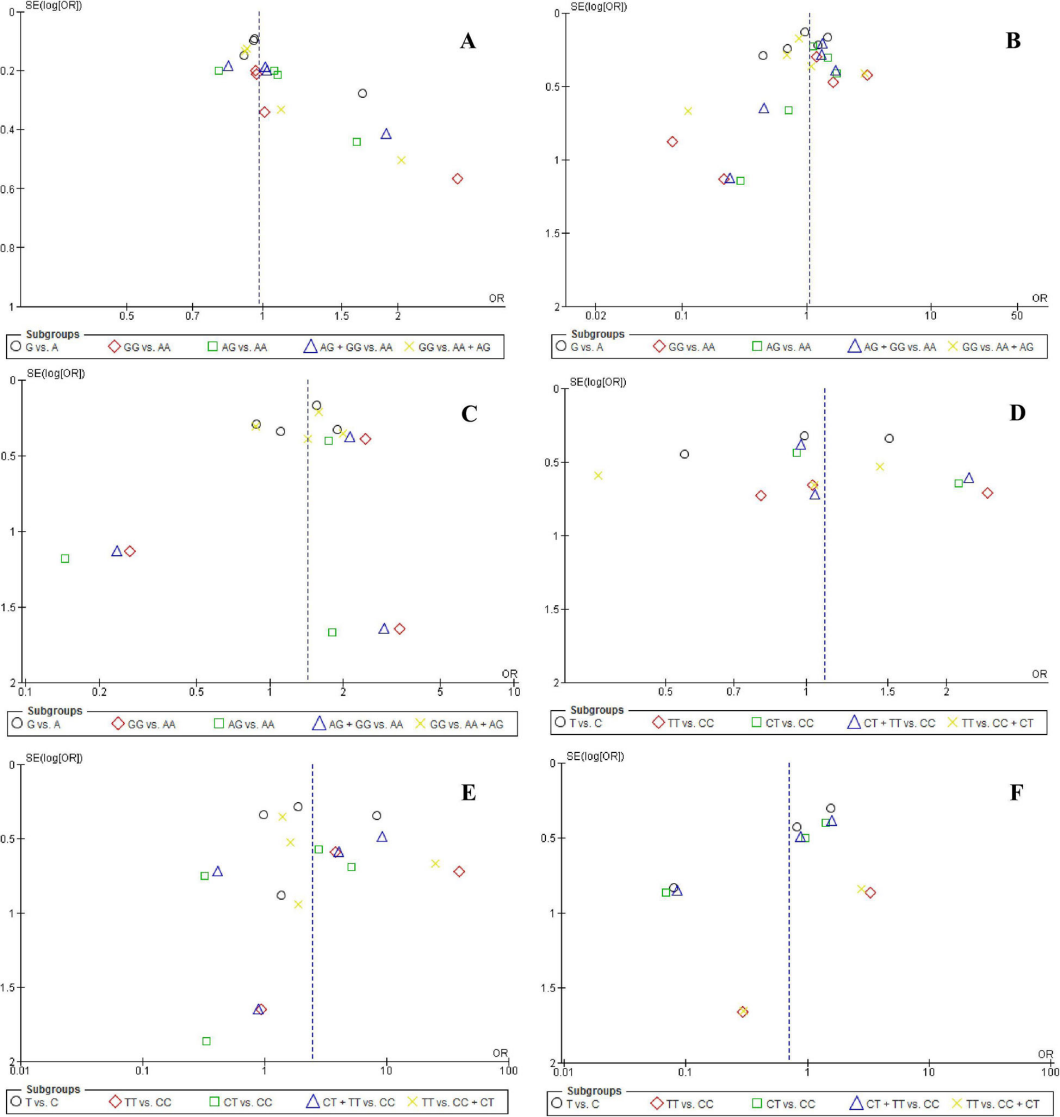
Funnel plots of the analysis of each polymorphism with a minimum of three studies**:** A) LTF rs1126478, B) ENAM s1264848, C) ENAM rs3796704, D) AMELX rs946252, E) AMELX rs17878486, and F) AMELX rs2106416

Both Egger’s and Begg’s tests were done on the previous pooled analyses with a minimum of three studies (Fig. 2). The results revealed a publication bias regarding GG vs. AA, AG vs. AA, and AG + GG vs. AA models of *LTF* rs1126478 polymorphism (Begg’s test: *P* < 0.05) and also T vs. C and CT + TT vs. CC models of *AMELX* rs2106416 polymorphism (Begg’s test: *P* < 0.05).

## Discussion

The effect of environmental risk factors [26] and genetic predisposition [27] on development of caries has been well identified. This meta-analysis assessed the association between the common polymorphisms of *LTF*, *ENAM*, and *AMELX* and the risk of dental caries. The findings showed that *ENAM* rs3796704 polymorphism had an increased risk in the case group compared with the control group especially in the Caucasian ethnicity and studies with caries-free individuals as the control group.

The prevalence of *LTF* polymorphisms differs among various ethnicities [28]. The genotypes related to LTF level are associated with decreased function and this may lead to decreased defense against infection with stronger stimuli for granulocyte activation and desorption, leading to greater LTF release [28]. Four studies [5, 16, 20, 24] included in this meta-analysis assessed *LTF* rs1126478 polymorphism. However, none of them found any association between this polymorphism and dental caries susceptibility. In addition, the subgroup analysis in this meta-analysis did not find any association between this polymorphism and dental caries risk. Thus, we can exclude *LTF* rs1126478 polymorphism as a risk factor for dental caries; however, more accurate confirmation of results requires further research through larger studies on different ethnicities.

AMELX is the most significant factor for development of normal enamel [17] and *AMELX* aberration predominantly causes mineralization defects and congenital disorders such as amelogenesis imperfecta [29]. Therefore, some researchers suggest that genetic variations contribute to structural changes of the enamel and may create more levels of mineral loss, bacterial extension or biofilm deposition [30]. In our meta-analysis on *AMELX* rs946252, *AMELX* rs17878486, *AMELX* rs6639060, and *AMELX* rs2106416 polymorphisms, none of them was associated with the risk of dental caries. However, the sensitivity analysis revealed that *AMELX* rs17878486 polymorphism could be a risk factor for dental caries but the ethnicity and type of selected controls were the effective factors on the association between *AMELX* rs17878486 polymorphism and risk of dental caries. In addition, two studies included in our meta-analysis [11, 15] showed an association between *AMELX* rs17878486 polymorphism and dental caries susceptibility. Therefore, it may point to the role of *AMELX* rs17878486 polymorphism in development of dental caries more than others.

*ENAM* gene may also play a role in enamel formation [31] and any change in genes that encode enamel proteins may lead to enamel malformation [32]. Among studies on *ENAM* polymorphisms included in this meta-analysis, one study on Saudi patients [25] reported an elevated risk of *ENAM* rs1264848 polymorphism and another study on Polish children [14] showed the protective role of *ENAM* rs1264848 polymorphism in dental caries, which was confirmed by Abbasoğlu et al., in their study on Turkish children [33]. Also, one study [25] reported an elevated risk of *ENAM* rs3796704 polymorphism and another study [5] showed an elevated risk of *ENAM* rs3796703 polymorphism in dental caries patients compared with controls. One research [22] reported that there were significant differences in the minor allele frequency between the Poles and Czechs populations. In addition, the sensitivity analysis confirmed the effect of ethnicity and type of control group on the association of *ENAM* rs3796704 and risk of dental caries. Bayram and colleagues [34] showed that *ENAM* polymorphisms may affect the development of enamel and these effects may be different between primary and permanent dentitions.

Small number of studies included in each analysis and different DMFT scores for selecting the cases and controls between the studies were two important limitations of the present meta-analysis. However, in most analyses, the heterogeneity was low and there was no publication bias.

## Conclusions

The findings of this meta-analysis confirmed that the G allele and the GG genotype of *ENAM* rs3796704 polymorphism were associated with an elevated risk of caries in the case group compared with the control group. But there was no association between *LTF* rs1126478, *ENAM* rs1264848, *ENAM* rs3796703, *AMELX* rs946252, *AMELX* rs17878486, and *AMELX* rs2106416 polymorphisms and dental caries susceptibility. However, subgroup analysis showed an association between *ENAM* rs3796704 and *AMELX* rs17878486 polymorphisms and dental caries susceptibility in the Caucasian ethnicity and studies including caries-free individuals as the control group. In addition, sensitivity analysis showed an increased risk of *AMELX* rs17878486 polymorphism in the case group compared with the control group. The future analyses with more cases in various areas should have focused on possible effects of gene-environmental interactions on caries experience.

## Supplementary information

**Additional file 1.**

## Data Availability

The datasets used and/or analysed during the current study available from the corresponding author on reasonable request.

## Abbreviations

LTF: Lactotransferrin
ENAM: Enamelin
AMELX: Amelogenin X
OR: Odds ratio
HWE: Hardy-Weinberg equilibrium
CI: Confidence interval
DMFT: Decayed, Missing, and Filled Teeth
