# Supplementary material for: Association of LTF, ENAM, and AMELX polymorphisms with dental caries susceptibility: a meta-analysis
Source: BMC Oral Health. 2020 May 6;20:132. doi: 10.1186/s12903-020-01121-7 (PMC7204276; doi:10.1186/s12903-020-01121-7)
Supplement: Supplementary file 1 — Additional file 1. [file 12903_2020_1121_MOESM1_ESM.pdf]

Study or Subgroup	Caries Events	Caries Total	Control Events	Control Total	Weight	Odds Ratio M-H, Fixed, 95% CI
**1.1.1 G vs. A**						
Azevedo, 2010	62	124	36	96	3.7%	1.67 [0.97, 2.87]
Volckova, 2014	238	964	82	310	17.0%	0.91 [0.68, 1.22]
Wang, 2017	660	1010	663	1000	41.9%	0.96 [0.80, 1.15]
Wang, 2018	664	1014	536	806	37.4%	0.96 [0.79, 1.16]
**Subtotal (95% CI)**		**3112**		**2212**	**100.0%**	**0.98 [0.87, 1.10]**
Total events	1624		1317			
Heterogeneity: Chi² = 4.03, df = 3 (P = 0.26); I² = 25%						
Test for overall effect: Z = 0.41 (P = 0.68)						
**1.1.2 GG vs. AA**						
Azevedo, 2010	16	32	7	26	3.3%	2.71 [0.89, 8.23]
Volckova, 2014	44	332	13	99	14.9%	1.01 [0.52, 1.96]
Wang, 2017	219	283	227	291	43.4%	0.96 [0.65, 1.43]
Wang, 2018	220	283	184	235	38.4%	0.97 [0.64, 1.47]
**Subtotal (95% CI)**		**930**		**651**	**100.0%**	**1.03 [0.80, 1.33]**
Total events	499		431			
Heterogeneity: Chi² = 3.12, df = 3 (P = 0.37); I² = 4%						
Test for overall effect: Z = 0.23 (P = 0.82)						
**1.1.3 AG vs. AA**						
Azevedo, 2010	30	46	22	41	5.3%	1.62 [0.68, 3.84]
Volckova, 2014	150	438	56	142	36.3%	0.80 [0.54, 1.18]
Wang, 2017	222	286	209	273	31.2%	1.06 [0.72, 1.58]
Wang, 2018	224	287	168	219	27.3%	1.08 [0.71, 1.64]
**Subtotal (95% CI)**		**1057**		**675**	**100.0%**	**1.00 [0.80, 1.25]**
Total events	626		455			
Heterogeneity: Chi² = 2.67, df = 3 (P = 0.44); I² = 0%						
Test for overall effect: Z = 0.01 (P = 0.99)						
**1.1.4 AG + GG vs. AA**						
Azevedo, 2010	46	62	29	48	4.8%	1.88 [0.84, 4.24]
Volckova, 2014	194	482	69	155	35.6%	0.84 [0.58, 1.21]
Wang, 2017	441	505	436	500	31.7%	1.01 [0.70, 1.47]
Wang, 2018	444	507	352	403	27.8%	1.02 [0.69, 1.52]
**Subtotal (95% CI)**		**1556**		**1106**	**100.0%**	**0.99 [0.81, 1.23]**
Total events	1125		886			
Heterogeneity: Chi² = 3.23, df = 3 (P = 0.36); I² = 7%						
Test for overall effect: Z = 0.05 (P = 0.96)						
**1.1.5 GG vs. AA + AG**						
Azevedo, 2010	16	62	7	48	2.2%	2.04 [0.76, 5.44]
Volckova, 2014	44	482	13	155	6.6%	1.10 [0.57, 2.10]
Wang, 2017	219	505	227	500	48.0%	0.92 [0.72, 1.18]
Wang, 2018	220	507	184	403	43.1%	0.91 [0.70, 1.19]
**Subtotal (95% CI)**		**1556**		**1106**	**100.0%**	**0.95 [0.80, 1.13]**
Total events	499		431			
Heterogeneity: Chi² = 2.66, df = 3 (P = 0.45); I² = 0%						
Test for overall effect: Z = 0.55 (P = 0.58)						

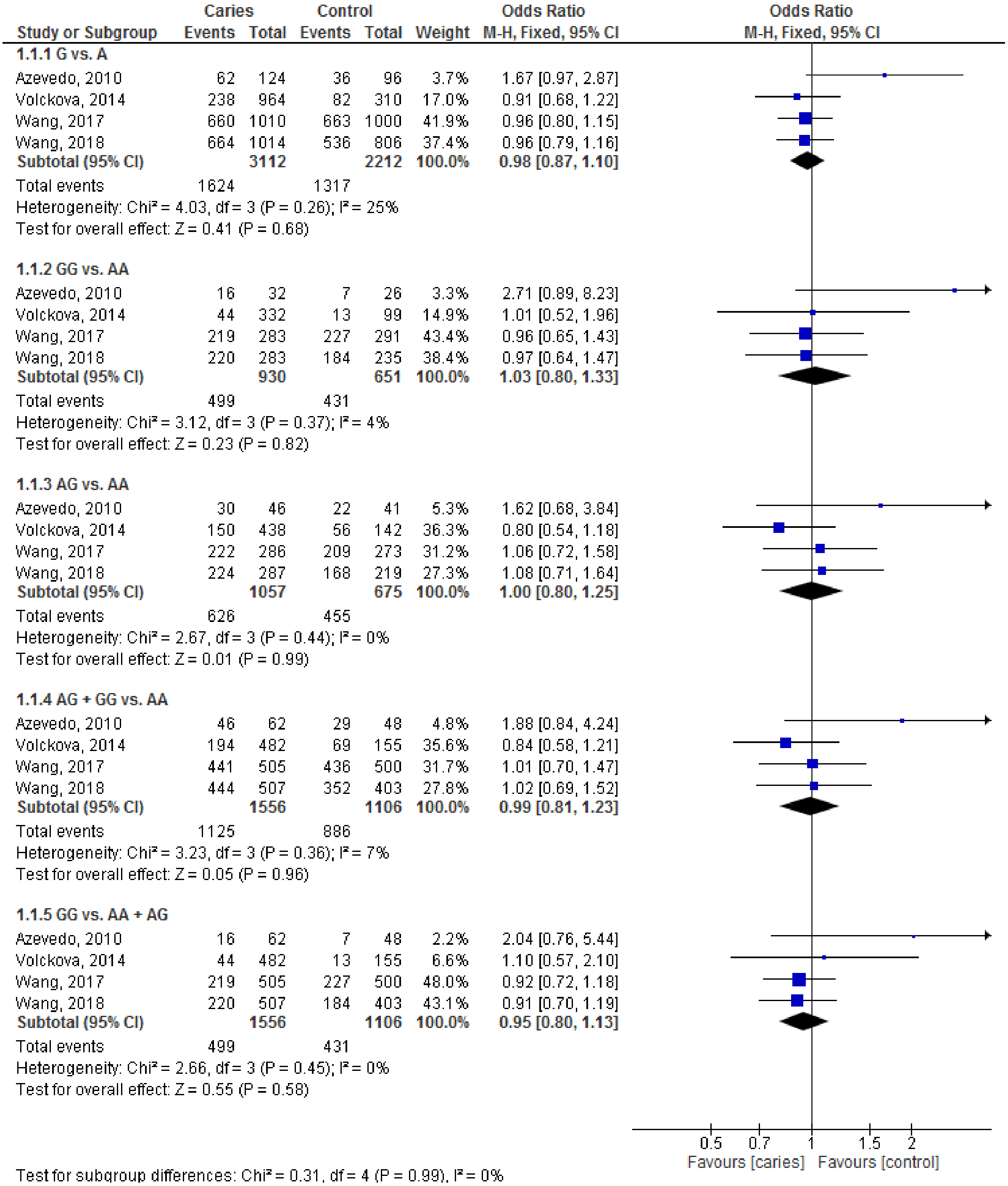

Test for subgroup differences: Chi² = 0.31, df = 4 (P = 0.99), I² = 0%

Figure 1: Forest plot of analysis of LTF rs1126478 polymorphism in high dental caries group compared to control group

Study or Subgroup	Caries Events	Caries Total	Control Events	Control Total	Weight	Odds Ratio M-H, Random, 95% CI
**1.2.1 G vs. A**						
Borilova Linhartova, 2018	733	1082	242	354	24.2%	0.97 [0.75, 1.26]
Devang Divakar, 2019	100	336	86	386	22.2%	1.48 [1.06, 2.07]
Ergöz, 2014	151	200	163	200	18.3%	0.70 [0.43, 1.13]
Gerreth, 2016	43	96	62	96	15.8%	0.44 [0.25, 0.79]
Jeremias, 2013	84	154	81	164	19.4%	1.23 [0.79, 1.91]
**Subtotal (95% CI)**		**1868**		**1200**	**100.0%**	**0.93 [0.65, 1.32]**
Total events	1111		634			
Heterogeneity: Tau² = 0.12; Chi² = 15.72, df = 4 (P = 0.003); I² = 75%						
Test for overall effect: Z = 0.41 (P = 0.68)						
**1.2.2 GG vs. AA**						
Borilova Linhartova, 2018	237	282	84	103	26.4%	1.19 [0.66, 2.15]
Devang Divakar, 2019	21	110	9	125	24.1%	3.04 [1.33, 6.96]
Ergöz, 2014	55	59	64	65	11.4%	0.21 [0.02, 1.98]
Gerreth, 2016	3	11	18	22	15.2%	0.08 [0.02, 0.46]
Jeremias, 2013	20	33	20	41	23.0%	1.62 [0.64, 4.09]
**Subtotal (95% CI)**		**495**		**356**	**100.0%**	**0.88 [0.34, 2.30]**
Total events	336		195			
Heterogeneity: Tau² = 0.82; Chi² = 16.78, df = 4 (P = 0.002); I² = 76%						
Test for overall effect: Z = 0.26 (P = 0.79)						
**1.2.3 AG vs. AA**						
Borilova Linhartova, 2018	259	304	74	93	23.5%	1.48 [0.81, 2.68]
Devang Divakar, 2019	58	147	68	184	51.3%	1.11 [0.71, 1.74]
Ergöz, 2014	41	45	35	36	4.8%	0.29 [0.03, 2.74]
Gerreth, 2016	37	45	26	30	7.8%	0.71 [0.19, 2.61]
Jeremias, 2013	44	57	41	62	12.6%	1.73 [0.77, 3.90]
**Subtotal (95% CI)**		**598**		**405**	**100.0%**	**1.21 [0.88, 1.65]**
Total events	439		244			
Heterogeneity: Chi² = 3.51, df = 4 (P = 0.48); I² = 0%						
Test for overall effect: Z = 1.17 (P = 0.24)						
**1.2.4 AG + GG vs. AA**						
Borilova Linhartova, 2018	496	541	158	177	25.1%	1.33 [0.75, 2.33]
Devang Divakar, 2019	79	168	77	193	48.0%	1.34 [0.88, 2.03]
Ergöz, 2014	96	100	99	100	5.0%	0.24 [0.03, 2.21]
Gerreth, 2016	40	48	44	48	9.3%	0.45 [0.13, 1.63]
Jeremias, 2013	64	77	61	82	12.6%	1.69 [0.78, 3.68]
**Subtotal (95% CI)**		**934**		**600**	**100.0%**	**1.24 [0.93, 1.67]**
Total events	775		439			
Heterogeneity: Chi² = 5.28, df = 4 (P = 0.26); I² = 24%						
Test for overall effect: Z = 1.45 (P = 0.15)						
**1.2.5 GG vs. AA + AG**						
Borilova Linhartova, 2018	237	541	84	177	25.3%	0.86 [0.61, 1.21]
Devang Divakar, 2019	21	168	9	193	19.0%	2.92 [1.30, 6.57]
Ergöz, 2014	55	100	64	100	22.5%	0.69 [0.39, 1.21]
Gerreth, 2016	3	48	18	48	12.8%	0.11 [0.03, 0.41]
Jeremias, 2013	20	77	20	82	20.4%	1.09 [0.53, 2.23]
**Subtotal (95% CI)**		**934**		**600**	**100.0%**	**0.83 [0.44, 1.58]**
Total events	336		195			
Heterogeneity: Tau² = 0.39; Chi² = 19.15, df = 4 (P = 0.0007); I² = 79%						
Test for overall effect: Z = 0.56 (P = 0.58)						

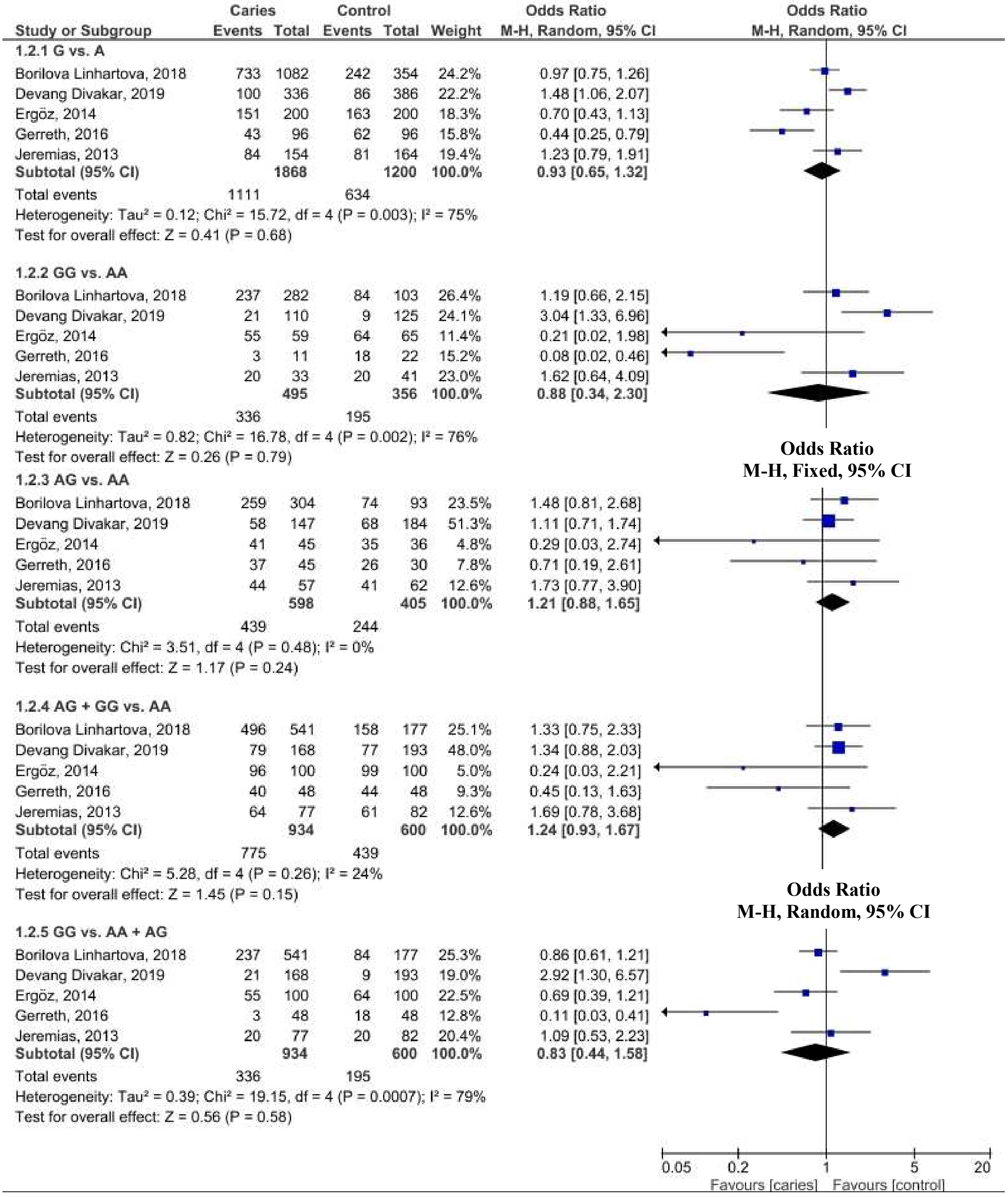

Figure 2: Forest plot of analysis of ENAM rs1264848 polymorphism in high dental caries group compared to control group

Study or Subgroup	Caries Events	Caries Total	Control Events	Control Total	Weight	Odds Ratio M-H, Fixed, 95% CI
**1.3.1 G vs. A**						
Devang Divakar, 2019	256	336	260	386	51.1%	1.55 [1.12, 2.16]
Ergöz, 2014	172	188	165	194	12.3%	1.89 [0.99, 3.61]
Jeremias, 2013	133	152	133	154	14.6%	1.11 [0.57, 2.15]
Koohpeima, 2018	440	472	312	332	22.0%	0.88 [0.49, 1.57]
**Subtotal (95% CI)**		**1148**		**1066**	**100.0%**	**1.38 [1.08, 1.76]**
Total events	1001		870			
Heterogeneity: Chi² = 4.13, df = 3 (P = 0.25); I² = 27%						
Test for overall effect: Z = 2.62 (P = 0.009)						
**1.3.2 GG vs. AA**						
Devang Divakar, 2019	99	110	92	117	68.1%	2.45 [1.14, 5.25]
Ergöz, 2014	78	78	69	70	3.5%	3.39 [0.14, 84.54]
Jeremias, 2013	61	65	57	58	28.3%	0.27 [0.03, 2.47]
Koohpeima, 2018	204	204	146	146		Not estimable
**Subtotal (95% CI)**		**457**		**391**	**100.0%**	**1.86 [0.96, 3.62]**
Total events	442		364			
Heterogeneity: Chi² = 3.55, df = 2 (P = 0.17); I² = 44%						
Test for overall effect: Z = 1.84 (P = 0.07)						
**1.3.3 AG vs. AA**						
Devang Divakar, 2019	58	69	76	101	66.6%	1.73 [0.79, 3.81]
Ergöz, 2014	16	16	27	28	4.0%	1.80 [0.07, 46.81]
Jeremias, 2013	11	15	19	20	29.4%	0.14 [0.01, 1.46]
Koohpeima, 2018	32	32	20	20		Not estimable
**Subtotal (95% CI)**		**100**		**149**	**100.0%**	**1.27 [0.64, 2.52]**
Total events	85		122			
Heterogeneity: Chi² = 4.03, df = 2 (P = 0.13); I² = 50%						
Test for overall effect: Z = 0.68 (P = 0.49)						
**1.3.4 AG + GG vs. AA**						
Devang Divakar, 2019	157	168	168	193	69.6%	2.12 [1.01, 4.46]
Ergöz, 2014	94	94	96	97	3.4%	2.94 [0.12, 73.03]
Jeremias, 2013	72	76	76	77	27.0%	0.24 [0.03, 2.17]
Koohpeima, 2018	236	236	166	166		Not estimable
**Subtotal (95% CI)**		**574**		**533**	**100.0%**	**1.64 [0.86, 3.14]**
Total events	559		506			
Heterogeneity: Chi² = 3.52, df = 2 (P = 0.17); I² = 43%						
Test for overall effect: Z = 1.50 (P = 0.13)						
**1.3.5 GG vs. AA + AG**						
Devang Divakar, 2019	99	168	92	193	43.3%	1.58 [1.04, 2.39]
Ergöz, 2014	78	94	69	97	14.2%	1.98 [0.99, 3.96]
Jeremias, 2013	61	76	57	77	13.8%	1.43 [0.67, 3.05]
Koohpeima, 2018	204	236	146	166	28.6%	0.87 [0.48, 1.59]
**Subtotal (95% CI)**		**574**		**533**	**100.0%**	**1.41 [1.06, 1.87]**
Total events	442		364			
Heterogeneity: Chi² = 3.65, df = 3 (P = 0.30); I² = 18%						
Test for overall effect: Z = 2.39 (P = 0.02)						

Test for subgroup differences: Chi² = 0.99, df = 4 (P = 0.91), I² = 0%

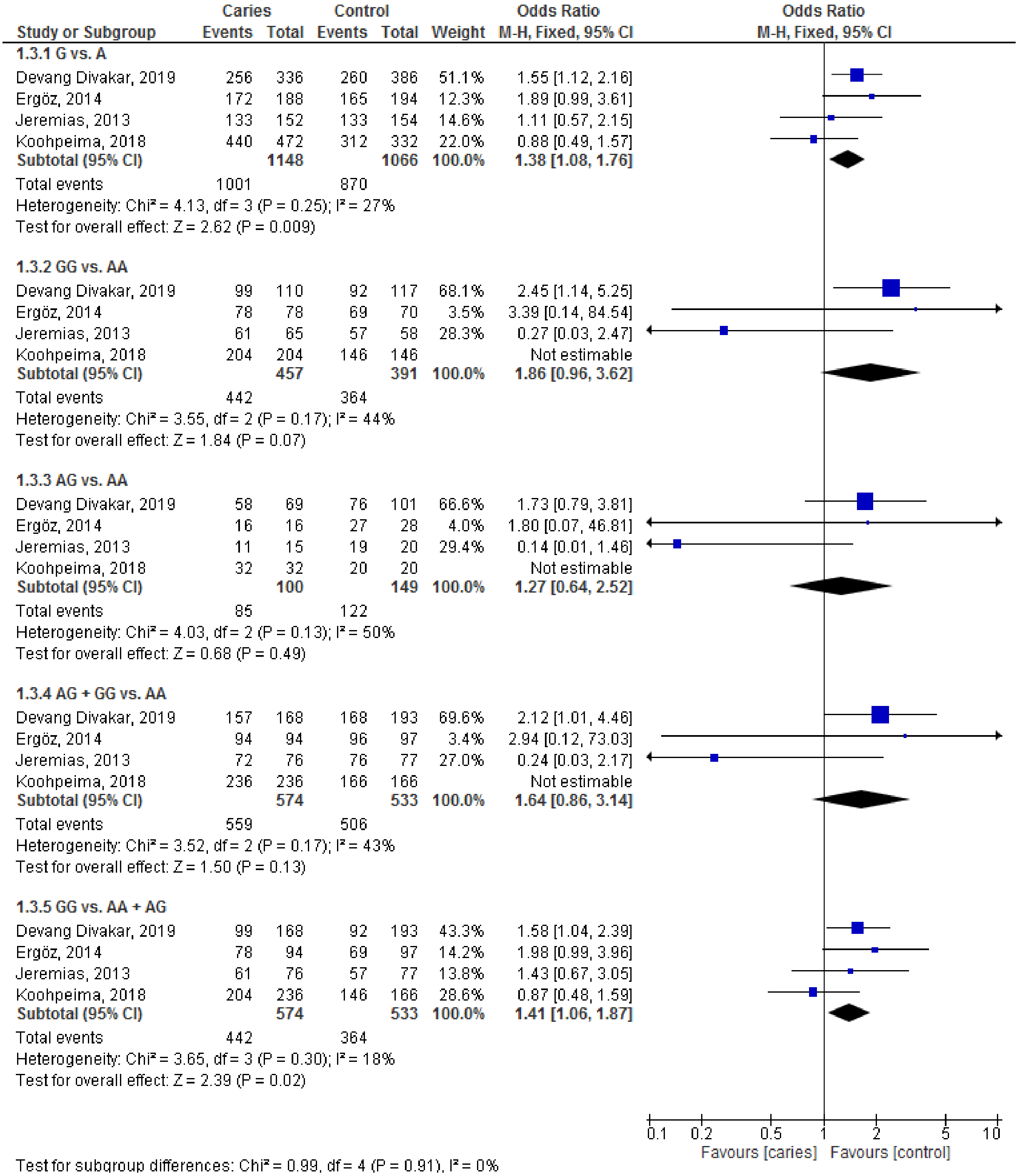

Figure 3: Forest plot of analysis of ENAM rs3796704 polymorphism in high dental caries group compared to control group

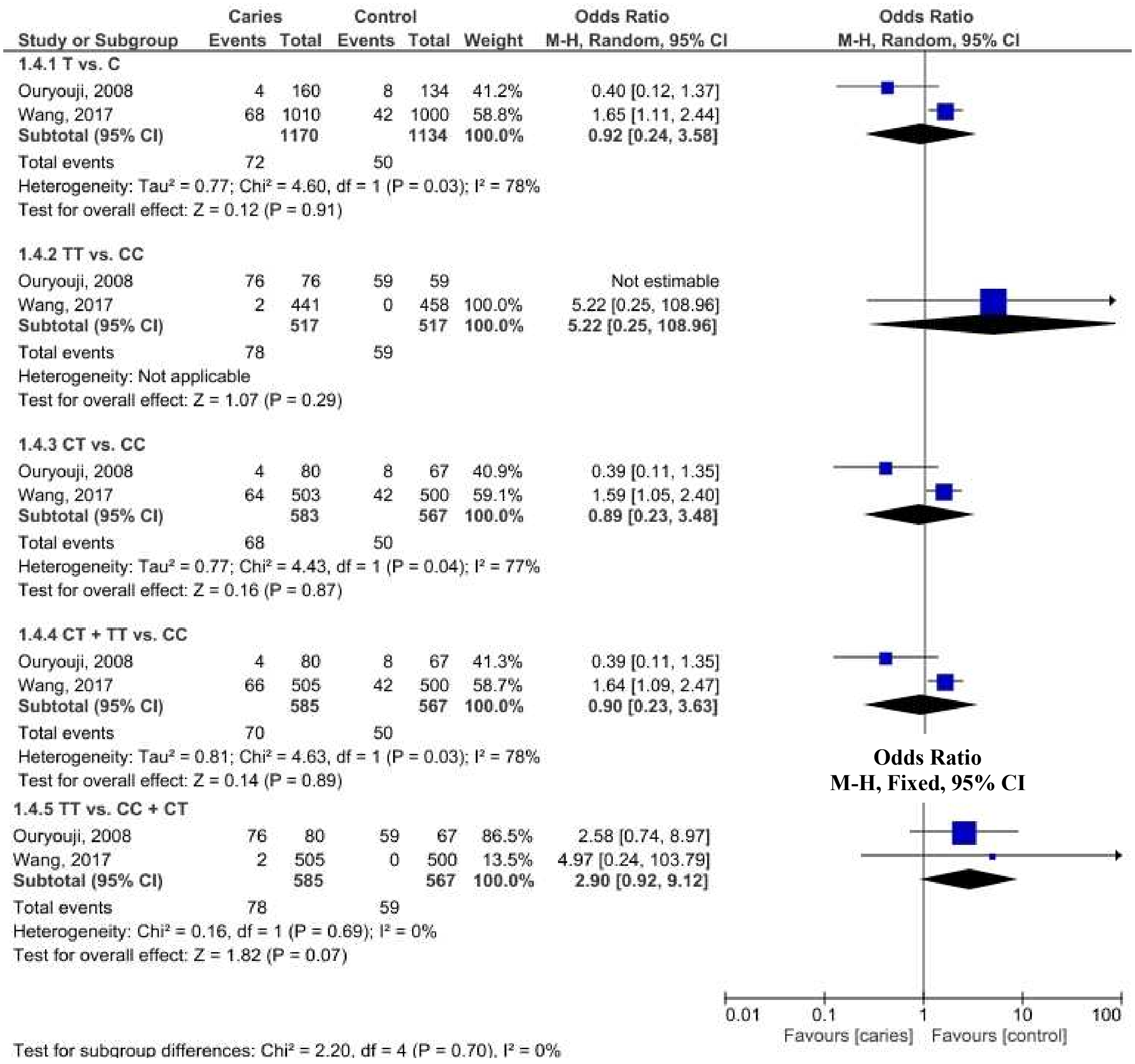

Figure 4: Forest plot of analysis of ENAM rs3796703 polymorphism in high dental caries group compared to control group

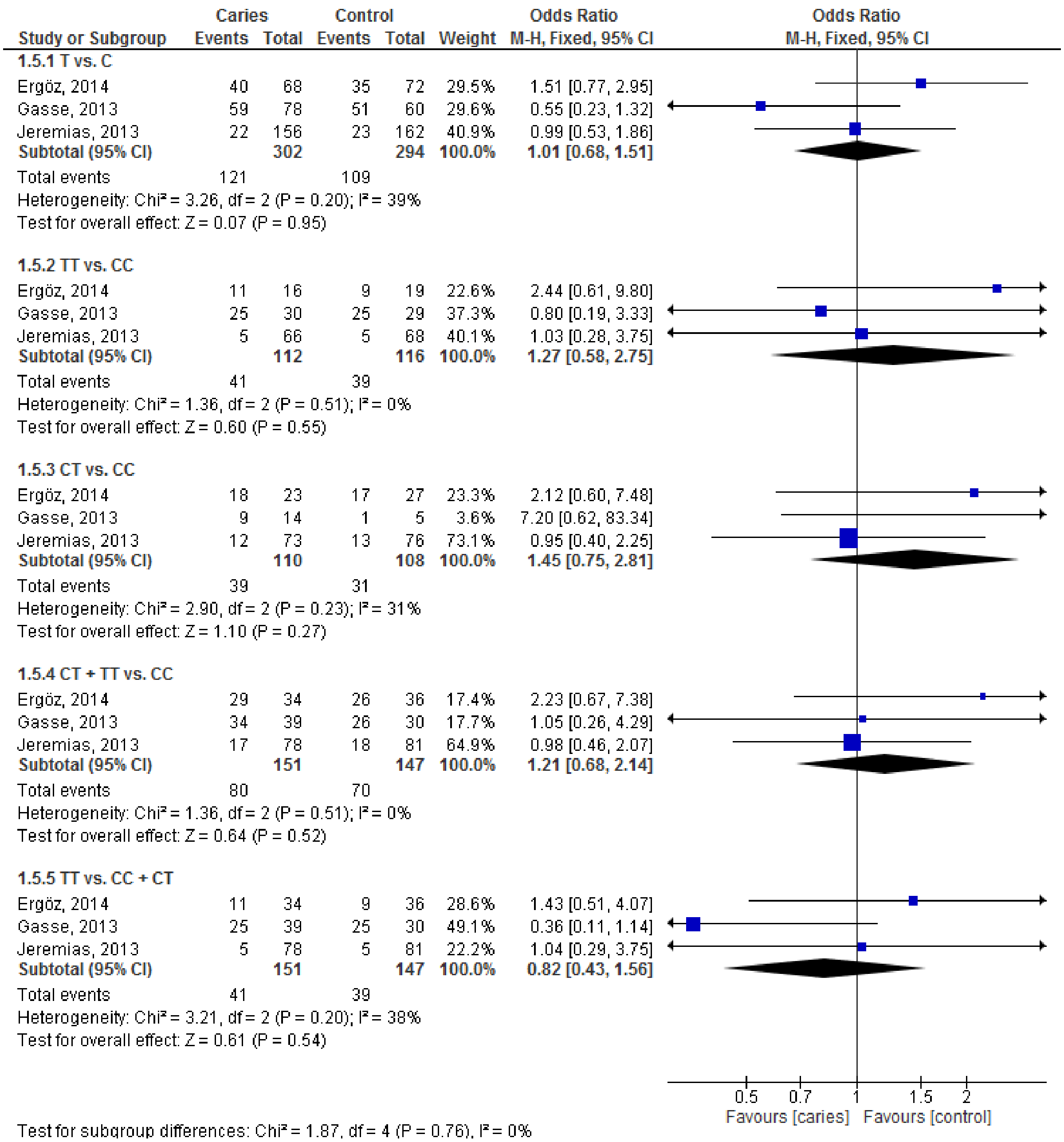

Figure 5: Forest plot of analysis of AMELX rs946252 polymorphism in high dental caries group compared to control group

Study or Subgroup	Caries Events	Caries Total	Control Events	Control Total	Weight	Odds Ratio M-H, Random, 95% CI
**1.6.1 T vs. C**						
Ergöz, 2014	46	80	37	64	27.6%	0.99 [0.51, 1.92]
Gerreth, 2017	70	96	20	82	27.5%	8.35 [4.25, 16.40]
Jeremias, 2013	129	152	123	164	28.5%	1.87 [1.06, 3.30]
Kang, 2011	166	170	60	62	16.5%	1.38 [0.25, 7.75]
**Subtotal (95% CI)**		**498**		**372**	**100.0%**	**2.25 [0.81, 6.24]**
Total events	411		240			
Heterogeneity: Tau² = 0.87; Chi² = 21.03, df = 3 (P = 0.0001); I² = 86%						
Test for overall effect: Z = 1.56 (P = 0.12)						
**1.6.2 TT vs. CC**						
Ergöz, 2014	14	22	8	11	26.6%	0.66 [0.13, 3.21]
Gerreth, 2017	30	38	3	34	27.6%	38.75 [9.38, 160.10]
Jeremias, 2013	57	61	56	71	29.2%	3.82 [1.19, 12.21]
Kang, 2011	82	83	29	29	16.6%	0.93 [0.04, 23.52]
**Subtotal (95% CI)**		**204**		**145**	**100.0%**	**3.59 [0.55, 23.32]**
Total events	183		96			
Heterogeneity: Tau² = 2.77; Chi² = 15.52, df = 3 (P = 0.001); I² = 81%						
Test for overall effect: Z = 1.34 (P = 0.18)						
**1.6.3 CT vs. CC**						
Ergöz, 2014	18	26	21	24	27.9%	0.32 [0.07, 1.40]
Gerreth, 2017	10	18	14	45	32.1%	2.77 [0.90, 8.51]
Jeremias, 2013	15	19	11	26	29.3%	5.11 [1.33, 19.72]
Kang, 2011	2	3	2	2	10.7%	0.33 [0.01, 12.82]
**Subtotal (95% CI)**		**66**		**97**	**100.0%**	**1.45 [0.36, 5.80]**
Total events	45		48			
Heterogeneity: Tau² = 1.23; Chi² = 9.00, df = 3 (P = 0.03); I² = 67%						
Test for overall effect: Z = 0.53 (P = 0.60)						
**1.6.4 CT + TT vs. CC**						
Ergöz, 2014	32	40	29	32	26.8%	0.41 [0.10, 1.71]
Gerreth, 2017	40	48	17	48	30.7%	9.12 [3.48, 23.87]
Jeremias, 2013	72	76	67	82	29.1%	4.03 [1.27, 12.75]
Kang, 2011	84	85	31	31	13.4%	0.89 [0.04, 22.53]
**Subtotal (95% CI)**		**249**		**193**	**100.0%**	**2.30 [0.51, 10.33]**
Total events	228		144			
Heterogeneity: Tau² = 1.67; Chi² = 13.29, df = 3 (P = 0.004); I² = 77%						
Test for overall effect: Z = 1.09 (P = 0.28)						
**1.6.5 TT vs. CC + CT**						
Ergöz, 2014	14	40	8	32	26.8%	1.62 [0.58, 4.53]
Gerreth, 2017	30	48	3	48	24.3%	25.00 [6.77, 92.34]
Jeremias, 2013	57	76	56	82	29.5%	1.39 [0.69, 2.80]
Kang, 2011	82	85	29	31	19.5%	1.89 [0.30, 11.85]
**Subtotal (95% CI)**		**249**		**193**	**100.0%**	**3.10 [0.85, 11.28]**
Total events	183		96			
Heterogeneity: Tau² = 1.35; Chi² = 15.71, df = 3 (P = 0.001); I² = 81%						
Test for overall effect: Z = 1.71 (P = 0.09)						

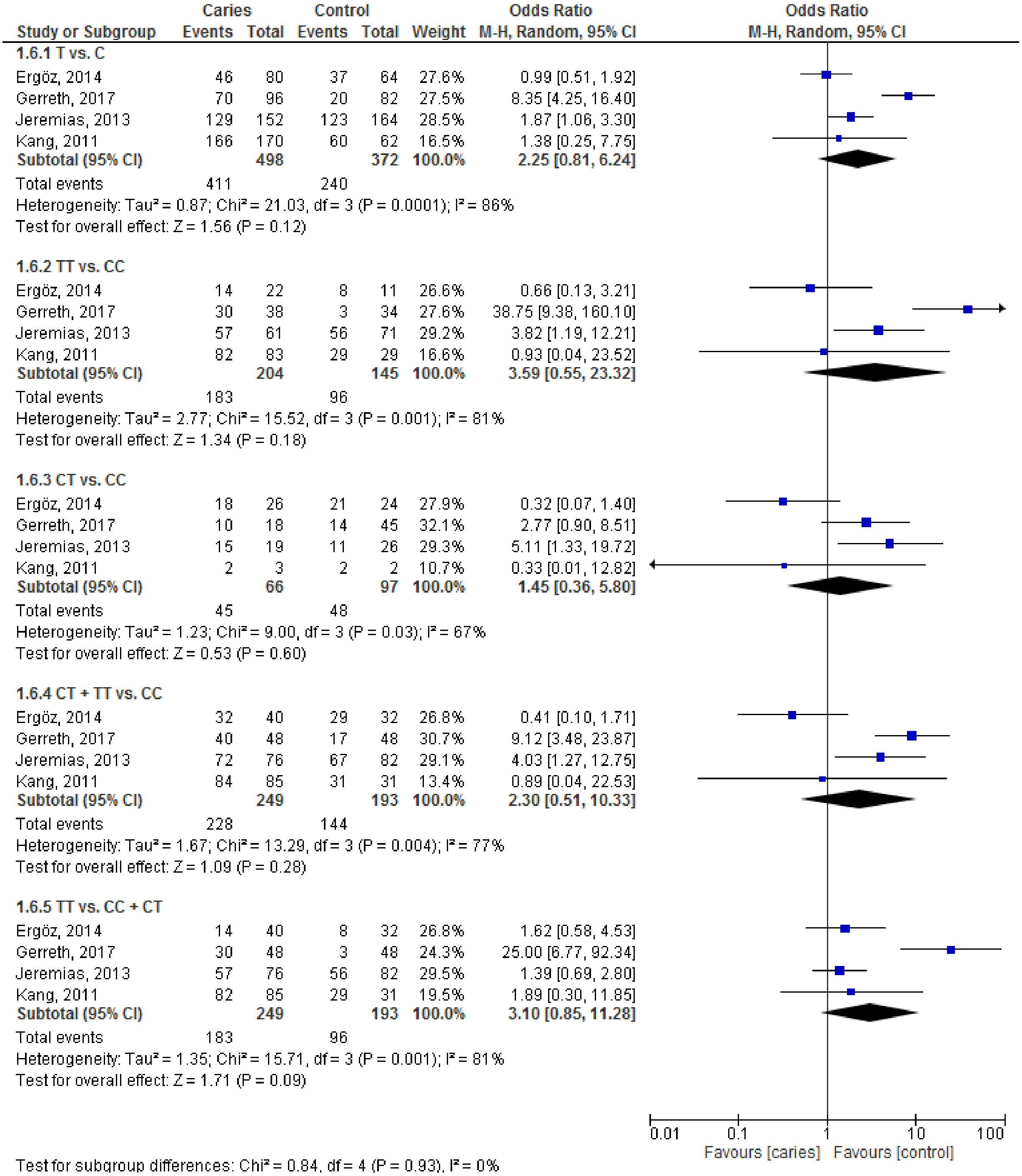

Test for subgroup differences: Chi² = 0.84, df = 4 (P = 0.93), I² = 0%

Figure 6: Forest plot of analysis of AMELX rs17878486 polymorphism in high dental caries group compared to control group

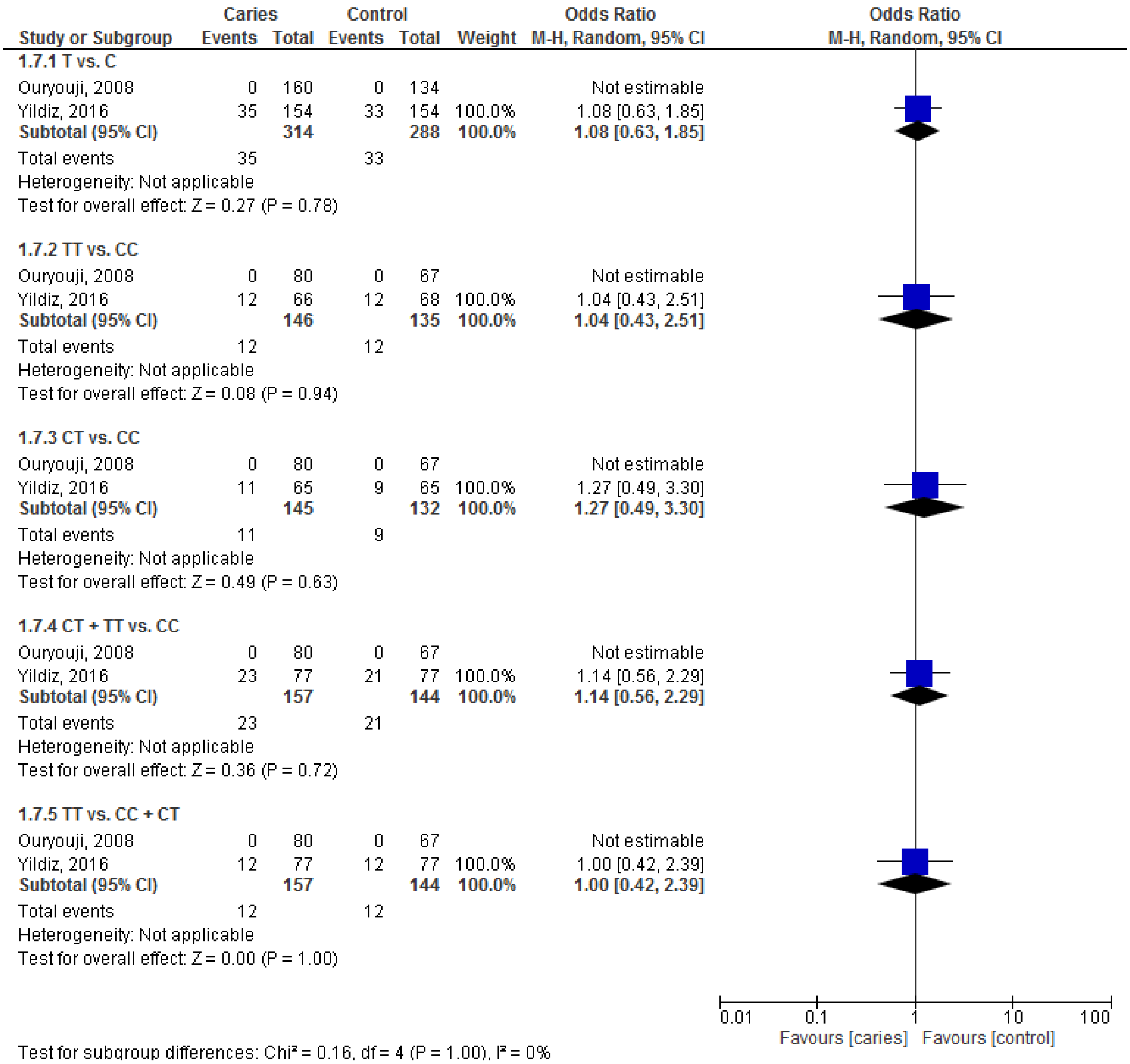

Figure 7: Forest plot of analysis of AMELX rs6639060 polymorphism in high dental caries group compared to control group

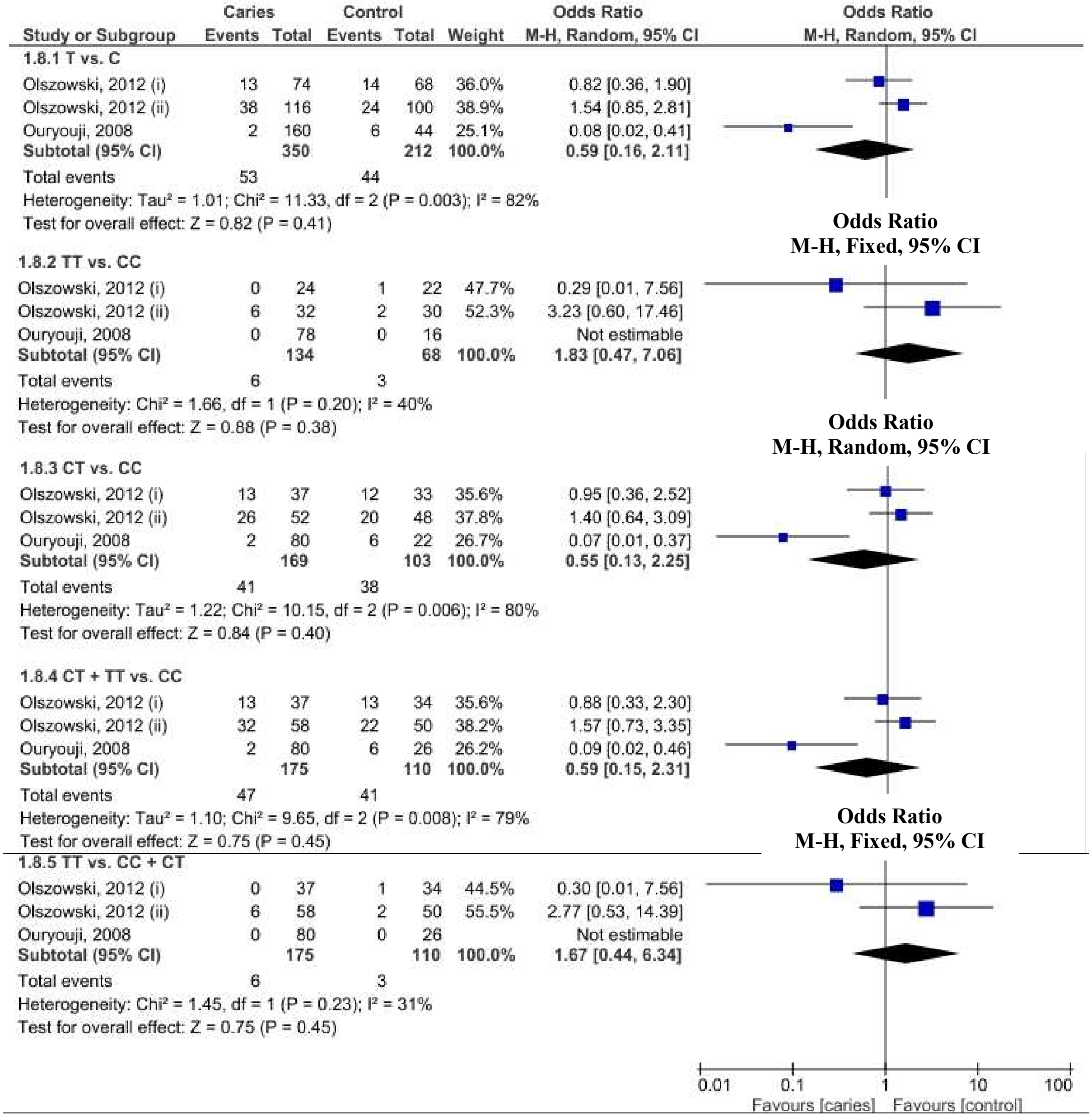

Figure 8: Forest plot of analysis of AMELX rs2106416 polymorphism in high dental caries group compared to control group
